# GMP production of 6-[^18^F]Fluoro-l-DOPA for PET/CT imaging by different synthetic routes: a three center experience

**DOI:** 10.1186/s41181-021-00135-y

**Published:** 2021-06-12

**Authors:** Valdemar L. Andersen, Mikkel A. Soerensen, Johan Hygum Dam, Niels Langkjaer, Henrik Petersen, Dirk Andreas Bender, Dan Fugloe, Tri Hien Viet Huynh

**Affiliations:** 1 Department of Nuclear Medicine, Copenhagen University Hospital Herlev and Gentofte, Borgmester Ib Juuls vej 31, DK-2730 Herlev, Denmark; 2 Department of Nuclear Medicine, Odense University Hospital, Odense, Denmark; 3 Department of Clinical Research, University of Southern Denmark, Odense, Denmark; 4 Department of Nuclear Medicine and PET Center, Aarhus University Hospital, Aarhus, Denmark

**Keywords:** [^18^F]FDOPA, Radiosynthesis, PET/CT imaging, GMP production

## Abstract

**Background:**

The radiofluorinated levodopa analogue 6-[^18^F]F-l-DOPA (3,4-dihydroxy-6-^18^F-l-phenylalanine) is a commonly employed radiotracer for PET/CT imaging of multiple oncological and neurological indications. An unusually large number of different radiosyntheses have been published to the point where two different Ph. Eur. monographs exist depending on whether the chemistry relies on electrophilic or nucleophilic radiosubstitution of appropriate chemical precursors. For new PET imaging sites wishing to adopt [^18^F]FDOPA into clinical practice, selecting the appropriate production process may be difficult and dependent on the clinical needs of the site.

**Methods:**

Data from four years of [^18^F]FDOPA production at three different clinical sites are collected and compared. These three sites, Aarhus University Hospital (AUH), Odense University Hospital (OUH), and Herlev University Hospital (HUH), produce the radiotracer by different radiosynthetic routes with AUH adopting an electrophilic strategy, while OUH and HUH employ two different nucleophilic approaches. Production failure rates, radiochemical yields, and molar activities are compared across sites and time. Additionally, the clinical use of the radiotracer over the time period considered at the different sites are presented and discussed.

**Results:**

The electrophilic substitution route suffers from being demanding in terms of cyclotron operation and maintenance. This challenge, however, was found to be compensated by a production failure rate significantly below that of both nucleophilic approaches; a result of simpler chemistry. The five-step nucleophilic approach employed at HUH produces superior radiochemical yields compared to the three-step approach adopted at OUH but suffers from the need for more comprehensive synthesis equipment given the multi-step nature of the procedure, including HPLC purification. While the procedure at OUH furnishes the lowest radiochemical yield of the synthetic routes considered, it produces the highest molar activity. This is of importance across the clinical applications of the tracer discussed here, including dopamine synthesis in striatum of subjects with schizophrenia and congenital hyperinsulinism in infants.

**Conclusion:**

For most sites either of the two nucleophilic substitution strategies should be favored. However, which of the two will depend on whether a given site wishes to optimize the radiochemical yield or the ease of the use.

**Supplementary Information:**

The online version contains supplementary material available at 10.1186/s41181-021-00135-y.

## Introduction

The fluoro-substituted levodopa analogue 6-fluoro-l-dopa (3,4-dihydroxy-6-fluoro-l-phenylalanine) is a substrate for l-amino acid decarboxylase (Firnau et al. 1980). The radiofluorinated version, 6-[^18^F]F-l-DOPA ([^18^F]**1**), initially developed as a means of visualizing intracerebral dopamine (Garnett et al. 1983a; Garnett et al. 1983b) using positron emission tomography (PET), has been used for PET/computed tomography (CT)-studies of the role of dopamine in various disorders such as chronic pain (Jääskeläinen et al. 2001), Asperger syndrome (Nieminen-von Wendt et al. 2004), schizophrenia (Elkashef et al. 2000), and Tourette’s disorder (Ernst et al. 1999). Furthermore, [^18^F]**1** has current clinical use in PET/CT studies of diseases involving malfunctions in the dopaminergic system and certain forms of cancer, e.g. Parkinson’s disease (Sioka et al. 2010), medullary thyroid carcinoma (Kushchayev et al. 2019), gastroenteropancreatic neuroendocrine tumors (Deroose et al. 2016), congenital hyperinsulinism (Blomberg et al. 2013), and gliomas (Xiao et al. 2019).

**Scheme 1:**
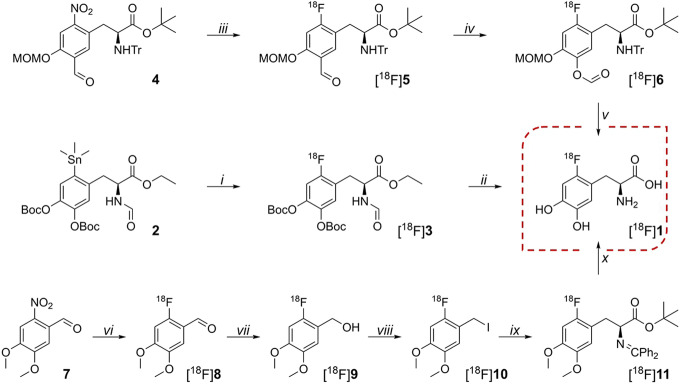
The three different synthetic pathways to [^18^F]1 discussed in the present work. *i*, [^18^F]F_2_, CHCl_3_, RT; *ii*, 57% HI, 150 °C; *iii*, [^18^F]TBAF, DMSO, 110 °C; *iv*, *m*-CPBA, MeCN, 55 °C; *v*, 30% HCl, EtOH, 50 °C; *vi*, [^18^F]KF/K_222_, DMF, 165 °C; *vii*, NaBH_4_, H_2_O @ tC18, RT; *viii*, 57% HI @ tC18, RT; *ix*, Ph_2_C=NCH_2_Boc, DCM, cPTC, KOH, − 20 °C; *x*, 57% HI, 165 °C.

[^18^F]**1** has indeed been hailed for its usefulness as a multi-target molecule in PET/CT-studies (Minn et al. 2009), but the clinical use has to some extent been hampered by cumbersome and/or complicated radiosynthetic procedures (Pretze et al. 2014). Synthesis of [^18^F]**1** was first achieved using [^18^F]XeF_2_ (Firnau et al. 1980; Chirakal et al. 1984), obtained from the ^20^Ne(d,α)^18^F nuclear reaction and subsequent reaction of the so-obtained carrier-added [^18^F]F_2_ with xenon. However, a radiochemical yield (RCY) of less than 1% prompted the same group to suggest an alternate method employing direct electrophilic fluorination of l-DOPA using [^18^F]F_2_ (Firnau et al. 1984). However, even with this alternate method the RCY was still a mere 3%, prompting the publication of a plethora of alternate and optimized electrophilic procedures in the ensuing decades (Pretze et al. 2014). Of the published electrophilic procedures, the fluorination of the commercially available enantiopure trimethylstannyl-precursor **2** (Scheme 1) with carrier-added [^18^F]F_2_ (Dolle et al. 1998) remains the most commonly employed, and it is this method that is used for routine clinical production of [^18^F]**1** at Aarhus University Hospital (AUH).

The main alternative to the electrophilic synthesis of [^18^F]**1** is by nucleophilic substitution of an appropriate nitro- or trimethylammonium-substituted precursor with [^18^F]F^−^ produced via the ^18^O(p,n)^18^F nuclear reaction (Pretze et al. 2014). Two main methods have been commercialized for automated synthesis. In one approach, the fully protected enantiopure nitro-precursor **4** (Scheme 1) is radiofluorinated using [^18^F]TBAF (TBA = tetra(*n*-butyl)ammonium), followed by a Baeyer-Villiger oxidation of the labelled aldehyde [^18^F]**5**, and global deprotection of the resulting formate ester [^18^F]**6** to furnish [^18^F]**1** (Wagner et al. 2009; Pretze et al. 2017). This method is employed for the routine clinical production of [^18^F]**1** at Odense University Hospital (OUH). The other approach is a multi-step synthesis starting with radiofluorination of nitroveratraldehyde **7** with [^18^F]KF/K_222_ (K_222_ = Kryptofix® 222 = 4,7,13,16,21,24-hexaoxa-1,10-diazabicyclo [8.8.8]hexacosane) as outlined in Scheme 1. Unlike the two methods described in the above, the stereocenter is introduced during the synthesis by performing an enantioselective alkylation in the presence of a chiral phase-transfer catalyst (Libert et al. 2013). It is this method which is used for the routine clinical production of [^18^F]**1** at Herlev University Hospital (HUH).

Recently, two different groups have published automated syntheses of 6-[^18^F]F-l-DOPA on the Tracerlab FX system utilizing the copper mediated fluorination of aryl pinacol boronates introduced by the Gouverneur’s group (Tredwell et al. 2014). This approach has resulted in isolated radiochemical yields in the 5–14.5% range (Mossine et al. 2020; Orlovskaya et al. 2020).

In this study, we compare the recent production results from the above-mentioned clinical sites (AUH: *n* = 97, OUH: *n* = 74, HUH: *n* = 135) and discuss the advantages and disadvantages of the synthetic procedures in question. Additionally, the differences in clinical use of [^18^F]**1** across the sites and the advantages and disadvantages of the different manufacturing approaches in relation to the clinical application will be discussed.

## Materials and methods

### Radiosynthesis of [^18^F]1

#### AUH

The cyclotron parameters used in the production of carrier-added [^18^F]F_2_ are summarized in Supplemental Table 1. The target gas ([^18^O]O_2_ > 98%) used for the first bombardment on the cyclotron is obtained from Campro Scientific (Berlin, Germany). The synthesis is carried out using a custom-build synthesis platform (see Supplemental Figs. 1 and 2). An overall synthesis flowchart can be found in Supplemental Fig. 3. The precursor **2** for radiolabeling is obtained from ABX advanced biochemical compounds (Radeberg, Germany), standard reagents are obtained from Sigma-Aldrich, sterile solutions from the Aarhus University Hospital Pharmacy (Aarhus, Denmark), SPE cartridges and sterile filters from Waters, and the high-performance liquid chromatography (HPLC) column is obtained from Phenomenex. The radiosynthesis is described in detail in the Supplemental Data. Following completed deprotection of [^18^F]**3** (see Scheme 1), the crude product is purified by semi-preparative radio-HPLC using a Spherisorb ODS column (250 × 10 mm) and sodium dihydrogen phosphate buffer (70 mm, sterile) as eluent at a flow rate of 3 ml/min. [^18^F]**1** elutes at 11–13 min and is collected directly into the final sterile product vial via a Millipore-GS 0.22 μm sterilizing filter.

#### OUH

No-carrier added fluorine-18 is prepared by standard proton irradiation of [^18^O]H_2_O (≥ 97% enrichment, Rotem Industries, Israel or ABX advanced biochemical compounds, Radeberg, Germany) on a GE Healthcare 16 MeV cyclotron equipped with niobium target bodies. The automated synthesis of [^18^F]**1** is performed on a GE Tracerlab MX module, and the cassettes, reagent kits, and sterile, sealed product vials are obtained from ABX advanced biochemical compounds (Radeberg, Germany). An overview of the Tracerlab MX layout within the synthesis unit control software, and an overall synthesis flowchart can be found in Supplemental Figs. 4 and 5. The contents of the different reagent vials are as follows (see Supplemental Fig. 6). Following a complete synthesis, the formulated product is collected into the final sterile product vial via a Cathivex-GV 0.22 μm sterilizing filter.

#### HUH

No-carrier added fluorine-18 is prepared by standard proton irradiation of [^18^O]H_2_O (≥ 98% enrichment, Rotem Industries, Israel or ABX advanced biochemical compounds, Radeberg, Germany) on an IBA Cyclone 18/18 cyclotron equipped with niobium target bodies. [^18^F]**1** is synthesized using the Trasis AllinOne (AiO) automated radiochemistry module, and the single-use cassettes and reagents are supplied by Trasis (Ans, Belgium). The Xbridge BEH C18 OBD Prep Column (130 Å, 5 μm, 10 mm × 250 mm) used for the semi-preparative HPLC-purification is obtained from Waters. An overview of the AiO layout within the synthesis unit control software and the setup for production of [^18^F]**1** can be found in Supplemental Fig. 7 and 8. Although the exact amounts of reagents are copyright of Trasis, the contents of the different reagent vials are summarized in Supplemental Fig. 8. Following completed deprotection of [^18^F]**11** (see Scheme 1), the crude product is purified by semi-preparative radio-HPLC using sterile sodium acetate buffer as eluent at a flow rate of 5 ml/min. The reagents needed for preparing the eluent are also included in the reagent kit. [^18^F]**1** elutes at 6–8 min and is collected and formulated directly into the final sterile product vial via a Millipore-GS 0.22 μm sterilizing filter.

### Product quality control and analyses

All batches released for human use at all three sites are subjected to quality control (QC) tests in accordance with the respective compassionate use permits issued by the Danish Medicines Agency. The analytical methods, used for the quality control, are generally validated according to the current European Pharmacopeia and International Conference on Harmonization guidelines. Although, Ph. Eur. monographs 1918 (01/2008) and 2481 (04/2019) pertain to the production of [^18^F]**1**, compassionate use permits were issued prior to inclusion of the relevant monographs in the pharmacopeia, and deviations from Ph. Eur. in the QC methods reflect this fact. Details on the QC procedures and detailed specifications of the drug products at each production site are described in the Supplemental Data (see Supplemental Table 2, 3 and 4, and Supplemental Fig. 9–13).

### Statistical methods

The data are generally presented as mean ± standard error of the mean. The statistical significance of different means was calculated by a two-sample *t*-test, while the statistical significance of the difference in production failure rates was assessed using the Pearson’s *χ*^2^-test. A *p*-value of ≤0.05 was defined as statistically significant.

### Ethical treatment of humans and animals

A subset of the clinical data presented and discussed in the present contribution were obtained as part of a clinical trial at HUH examining presynaptic dopamine synthesis capacity in the striatum in antipsychotic-naïve patients with first-episode psychosis (The Ethics Committee of Capital Region of Denmark: H-3-2013-149). The remainder of the clinical data were retrieved from the database of existing patient PET/CT scans obtained as part of routine clinical practice. Additionally, the diagnoses are sufficiently common for specific scans to be discussed without compromising the privacy and confidentiality of the subjects in question. These considerations warrant the absence of ethics committee/review board approval.

## Results

The three different approaches to the synthesis of [^18^F]**1** are summarized in Scheme 1. The electrophilic method employed at AUH involves production of [^18^F]F_2_ from the ^18^O(p,n)^18^F nuclear reaction followed by recovery of the radiofluorine by a second proton bombardment on a noble gas-natural fluorine mixture (0.5–1% F_2_ in either Ar or Ne, see Supplemental Table 1 for details). The carrier-added [^18^F]F_2_, obtained from this “double-shoot” method (Nickles et al. 1984; Nickles et al. 1983), is transferred to a custom-build automated synthesis platform (see Supplemental Fig. 1) where it is passed through a chloroform solution of trimethylstannyl-precursor **2** (10 mg/ml). The subsequent hydrolysis of radiofluorinated [^18^F]**3** proceeds in concentrated hydroiodic acid. Following hydrolysis, the crude reaction mixture is partially neutralized with aqueous NaOH (3 m) before being purified by reversed phase semi-preparative HPLC. Sterile phosphate buffer (70 mm) is employed as eluent, and [^18^F]**1** elutes after 11–13 min. A total of 10 ml of eluate is collected and sterilized by filtration (0.22 μm) into the final sterile product vial. Thus, the sterile phosphate buffer used for product elution also serves as product formulation. [^18^F]**1** is obtained in a non-decay corrected (n.d.c.) RCY of 9.3 ± 4.2%, 45 min from the end of the second bombardment. Relevant production and QC parameters are summarized in Table 1.

**Table 1:** Average values of selected production and QC parameters for batches of [^**18**^F]**1** produced at the three different productions sites. Errors indicate standard deviations of the mean. The value for the enantiomeric purity at HUH includes productions where the alkylation is carried out at − 20 °C and at room temperature (rt)

	AUH	OUH	HUH
non-d.c. RCY	9.3 ± 4.2%	5.6 ± 0.3%	30 ± 8%
Radiochemical purity	97.4 ± 1.4%	98.4 ± 0.16%	99.8 ± 0.5%
[^18^F]F^−^	1.06 ± 0.84%	0.74 ± 0.1%	~ 0.2%^a^
Enantiomeric purity (l-isomer)	99.4 ± 0.2%	99.9 ± 0.06%	98.6 ± 0.4% (− 20 °C)
			96.7 ± 0.8% (rt)
Molar activity	52 ± 15 MBq/μmol	236 ± 28 GBq/μmol	189 ± 56 GBq/μmol
[^19^F]**1**	1.42 ± 0.68 mg/ml	0.32 ± 0.056 μg/ml	0.60 ± 0.13 μg/ml
6-OH-DOPA	3.84 ± 1.6 μg/ml	3.6 ± 0.18 μg/ml	Not detected

^a^The standard deviation of the mean is larger than the mean itself, making the value statistically insignificant

The nucleophilic substitution approach adopted for manufacturing of [^18^F]**1** at OUH relies on the production of [^18^F]F^−^ from the ^18^O(p,n)^18^F nuclear reaction in oxygen-18 enriched water. Following bombardment, the irradiated target water is transferred to a TRACERLab MX synthesis module, where the [^18^F]F^−^ is isolated, purified, and solvent exchanged on a QMA SPE cartridge mounted on a commercially available single-use cassette. Dry [^18^F]TBAF is obtained after elution with TBAHCO_3_ in acetonitrile followed by azeotropic drying and is subsequently reacted with nitro-precursor **4** in DMSO furnishing the radiolabeled aldehyde [^18^F]**5** by *ipso* displacement of the nitro moiety. The crude aldehyde is purified with water on a Chromafix C18ec cartridge before being eluted with MeCN. A subsequent Baeyer-Villiger oxidation of the aldehyde moiety using *meta*-chloroperoxybenzoic acid (*m*-CBPA) in MeCN furnishes formate ester [^18^F]**6**, which is converted into [^18^F]**1** by acid hydrolysis in a mixture of hydrochloric acid and ethanol. The crude mixture is diluted with phosphate-buffered saline (PBS), containing ethylenediaminetetraacetic acid (EDTA) and sodium ascorbate as stabilizers, filtered through a Sep-Pak C18 cartridge, and then trapped on a Chromafix HR-P cartridge. Fractionated elution of the cartridge with PBS (containing EDTA and sodium ascorbate) furnishes [^18^F]**1** in 24 ml of buffer, and the fraction is passed through Sep-Pak WAX and Alumina Light cartridges before being sterilized by filtration (0.22 μm) into the final sterile product vial. [^18^F]**1** is obtained in a non-decay corrected RCY of 5.6 ± 0.3%, 95 min from EOB. Relevant production and QC parameters are summarized in Table 1.

At HUH, the production of [^18^F]F^−^ is equivalent to that described at OUH. Following bombardment, the irradiated target water is transferred to a Trasis AiO synthesis module, where the [^18^F]F^−^ is isolated on a Sep-Pak QMA cartridge mounted on a commercially available single-use cassette. Dry [^18^F]KF/K_222_ is obtained after elution of the QMA with K_2_CO_3_/K_222_ in methanol followed by azeotropic drying and is subsequently reacted with nitroveratraldehyde **7** in DMF at elevated temperatures. The obtained radiolabeled benzaldehyde [^18^F]**8** is then trapped on a Sep-Pak tC18 cartridge and reduced to the corresponding benzyl alcohol [^18^F]**9** using aqueous NaBH_4_ directly on the cartridge. Subsequent iodination with concentrated aqueous HI affords radiolabeled benzyliodide [^18^F]**10**, simply by passing the acid slowly through the tC18 cartridge. [^18^F]**10** is eluted from the cartridge with DCM, and the eluate is dried by passing it through two consecutive SPE cartridges packed with K_2_CO_3_. The protected fluoro-substituted levodopa analog [^18^F]**11** is then obtained by the O’Donnell amino acid synthesis (O'Donnell et al. 1978), using the Schiff base *N*-(diphenylmethylene)glycine *tert*-butyl ester in the presence of a chiral phase-transfer catalyst and KOH. Subsequent acid hydrolysis in concentrated hydroiodic acid furnishes crude [^18^F]**1**. The crude product is purified by reversed phase semi-preparative HPLC, using a sodium acetate buffer containing EDTA and ascorbic acid as stabilizers. [^18^F]**1** elutes at 6–10 min (varies from column to column) and 5 ml of eluate is collected and passed through a sterilizing filter (0.22 μm) into a sterile product vial containing a mixture of citrate buffer and ascorbic acid (20 ml). [^18^F]**1** is obtained in a non-decay corrected RCY of 30 ± 8%, 70 min from EOB. Relevant production and QC parameters are summarized in Table 1.

## Discussion

The production of [^18^F]**1** in multidose quantities is viable using either of the three synthetic pathways discussed here. However, given their different nature, the challenges associated with the three production methods are rather different.

The nucleophilic methods rely on the production of fluorine-18 in a [^18^O]H_2_O-filled liquid target via the ^18^O(p,n)^18^F nuclear reaction. The handling of the target material, as well as the target itself, is straightforward, and such setups generally deliver stable performances over long periods. The generation of radiofluorine gas for the electrophilic method on the other hand requires significantly more care and maintenance. While the nuclear reaction itself is the same, the target needs both pre- and post-bombardment treatment. After longer standstill or target maintenance, the Al targets require passivation by irradiation of gas mixtures containing natural fluorine gas. Prior to filling the target with [^18^O]O_2_, the target is optionally flushed with Ne and then evacuated for 30 min (see Supplemental Table 1 for details). The [^18^O]O_2_-filled target is then subjected to proton irradiation for 60 min. The produced fluorine-18 is adsorbed on the walls of the target and following a completed bombardment, [^18^O]O_2_ can be recovered from the target using a cryotrap. In order to purify the recovered target gas of [^18^O]H_2_O, unavoidably produced during target irradiation, a 4 Å molecular sieve trap is placed between the target and the cryotrap. The produced radioactivity is recovered by a second, shorter proton bombardment on a noble gas-natural fluorine mixture (0.5–1% F_2_ in either Ar or Ne, see Supplemental Table 1 for details). However, if the molecular sieve trap has exceeded its capacity, the recirculated target gas will contain traces of water, and as a result only small amounts of non-reactive ^18^F radioactivity is delivered to the synthesis unit. As the presence of water furthermore degrades the target Al-F passivation, regular replacement of the molecular sieve trap is of utmost importance. Finally, the noble gas-fluorine gas mixture has a limited shelf life of one year, as the F_2_ content slowly deteriorates.

**Fig. 1:**
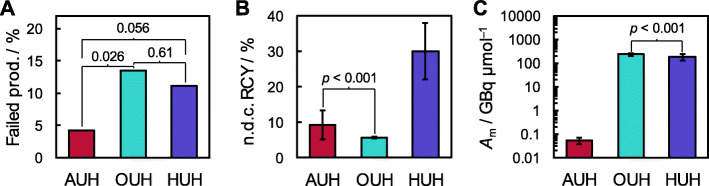
Production parameters for the three radiopharmaceutical production sites. **A** Percentage of failed productions. **B** Average non-decay corrected RCYs. **C** Average molar activity during the time period considered (note the log-scale on the ordinate). In (**A**) the numbers indicate the calculated *p*-values. In (**B**) and (**C**), a *p*-value in only given for the least different datasets

The above considerations regarding target maintenance and care seem to detract from the feasibility of the electrophilic pathway. However, the chemical simplicity of the electrophilic substitution reaction and subsequent deprotection results in a production failure rate significantly below that observed for both nucleophilic approaches, see Fig. 1A. The relative high failure rate of the cartridge-based nucleophilic method, employed at OUH, is in part due to a recurrent deviation where the trapping on the HR-P cartridge failed. This cartridge serves to separate the crude [^18^F]**1** from impurities while concomitantly removing excess hydrochloric acid employed in the hydrolysis step (Huang et al. 2020), and as a result, these failed batches suffered from very low yield and very acidic pH (1.8–2.6). This issue is expected to be remedied by transfer of the synthesis to the GE FASTlab platform. Of the only nine batches produced on the GE FASTlab and released for clinical application, none has failed, and the synthesis has delivered 9.9 ± 1.2% non-decay corrected radiochemical yield. This holds promise of a more robust synthesis and represents an improved yield over the Tracerlab MX synthesis and compares well to the yield by the electrophilic route at AUH. Despite the cartridge-related problem of the method, the use of cartridges for purification of the radiopharmaceutical, as opposed to the HPLC purification employed in the methods at both AUH and HUH, certainly counts as an advantage from the point-of-view of equipment maintenance and in compliance with good manufacturing practice (GMP).

**Fig. 2:**
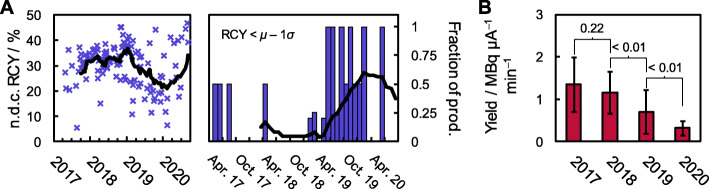
Temporal evolution of the radiochemical yields at HUH and AUH. **A** Non-decay corrected RCYs for the individual productions of [^18^F]1 at HUH as function of production date (left), and the fraction of the productions in any given month furnishing RCYs more than one standard deviation below the mean over the entire time period considered (right). In both figures, the bold line gives the moving average with a period of 12. **B** The annual mean radiochemical yield of [^18^F]1 at AUH given in units of MBq μA min^− 1^ in order to compare productions with differences in target current and irradiation time. The numbers indicate the calculated *p*-values

As for the nucleophilic substitution method employed at HUH, the main reason for failed productions was out-of-specifications (OOS) on the radioactivity concentration end-of-synthesis. Permitted daily exposure considerations, regarding the concentrated citrate buffer used as product formulation, enforces a lower limit of 111 MBq/ml, equivalent to 2.78 GBq of product. The main loss of activity, resulting in low yield and possible OOS, happened during the synthetic steps *ix* and *x* (see Scheme 1), namely the alkylation and the subsequent acid hydrolysis. During the automated cassette pre-treatment (i.e. prior to receiving any activity) dichloromethane (DCM) is added to the vial containing the solid reagents for the alkylation reaction (Supplemental Fig. 14) to dissolve the contents. It was discovered that the addition of the solvent tends to be rather vigorous, which at times results in suspended solids sticking to the bottom of the vial (the vial is upside down when mounted on the cassette). In some cases, this led to an incomplete addition of reagents during the alkylation step, and as a result, a large fraction of radioactivity would remain in reactor 2 after the transfer to the HPLC injection loop. As the crude reaction mixture is diluted with WFI prior to injection into the loop, it is suspected that the incomplete alkylation reaction leads to one or more water-insoluble radiolabeled byproducts. The problem can be reliably remedied by gently tapping the alkylation vial following addition of DCM. Notwithstanding this manual operation, the inter-operator variation in RCYs is negligible, and the four main operators at HUH are within error indistinguishable in this respect. Despite having relieved the problem of incomplete alkylation, the RCYs, on average, declined steadily from the first quarter of 2019 onwards as seen in Fig. 2A. This problem turned out to be rooted in deterioration of the build-in vacuum pump of the Trasis AiO system. Indeed, the chemical conditions employed in the preparation of [^18^F]**1** are significantly harsher than those employed to produce many common radiotracers, and the chemical wear and tear on the reusable parts of the system is consequently significant. The problem worsened through 2019 and into 2020, where more than half of all productions produced yields at least one standard deviation below the mean during the full production period. Replacement of the vacuum pump during the third quarter of 2020 resolved the issue, as seen by the sharp increase in average RCY in Fig. 2A.

While occasionally producing low yields, the average RCY of the multistep nucleophilic method employed at HUH is significantly higher than the two other methods discussed here (see Fig. 1B). However, the RCY of the electrophilic method used at AUH is reduced compared to its maximum practical potential (~ 25% non-decay corrected) for a couple of technical reasons. The initial procedure prescribes CFCl_3_ (i.e. Freon 11) as the reaction solvent (Dolle et al. 1998) for the fluorination reaction. As Freon was identified as major source for reduction of the ozone layer in 1990s, its sale was already limited when production of [^18^F]**1** at AUH commenced in 1997. Chloroform was found to be a viable substitute, although a loss of up to 25% of the incoming activity might be observed during trapping in this solvent. Subsequently, the major loss of radioactivity occurs on the Sep-Pak silica classic cartridges, placed between the fluorination and hydrolysis vessels (see Supplemental Figs. 1 and 2 for details), reflecting the formation of [^18^F]F^−^ during the fluorination reaction. Another loss is associated with the acid hydrolysis step. Despite extended reaction times (20 min) at elevated temperatures, the hydrolysis does not reach completion, with only ~ 80% [^18^F]**3** being converted into [^18^F]**1**. It is suspected that the somewhat uncommon use of hydroiodic acid for hydrolysis of Boc-protection groups to be the source of the incomplete conversion. However, 57% HI was found necessary to hydrolyze the phenolic O-methyl groups of the mercury precursor originally used in the manufacturing of [^18^F]**1** at AUH (Adam and Jivan 1988), and in order to limit the number of changed parameters associated with the change variation submitted to the Danish Medicines Agency upon substituting **2** for the mercury precursor, it was decided to leave the hydrolysis medium unchanged. Additionally, the yield suffered significantly from a change in production facility during 2019 with a concomitant increase in distance from the cyclotron to the GMP production laboratory (see Fig. 2B). This stresses the sensitivity of the gas target setup, as compared to the liquid targets employed for the nucleophilic approaches.

While superior in terms of RCY, the nucleophilic method employed at HUH suffers from the weakness that the stereocenter is introduced during the synthesis and controlling the enantiopurity of the [^18^F]**1** drug product is consequently crucial. While the chiral phase-transfer catalyst employed in alkylation step strongly favors the formation of levodopa analogue [^18^F]**1**, a smaller amount of the dextrodopa analogue 6-[^18^F]F-d-DOPA will form during the reaction (see Supplemental Fig. 13). If the alkylation was carried out at ambient temperature, an enantiomeric purity of 96.7 ± 0.8% (*n* = 13) was obtained, in excess of the specification limit of ≥95% in place at HUH. However, it was discovered that cooling the reaction mixture to − 20 °C using an acetone/dry ice bath increased the enantioselectivity of the alkylation thereby furnishing an enantiomeric purity of 98.6 ± 0.4% (*n* = 107). With the current European Pharmacopoeia requirement an enantiomeric purity of ≥95% is sufficient. The methods employed at AUH and OUH on the other hand utilize enantiopure precursors **2** and **4**, respectively, and given a sufficient purity of the starting material, obtaining the necessary enantiomeric purity of produced [^18^F]**1** is a non-issue. The use of enantiopure precursors has the added advantage of simplifying the QC procedure as only a single HPLC analysis needs to be completed prior to parametric release, as opposed to the two separate HPLC analyses carried out routinely for every batch at HUH.

The molar activities of [^18^F]**1** obtained from the nucleophilic methods are naturally vastly superior to that of the electrophilic one, given the carrier-added nature of the latter. The difference between the two approaches is roughly a factor of 4000 (see Fig. 1C), with the average molar activity observed at AUH being 52 ± 15 MBq/μmol. Interestingly, the molar activities at OUH and HUH both fall in the hundreds of GBq/μmol range (236 ± 28 GBq/μmol and 189 ± 56 GBq/μmol, respectively). Although the difference is significant (see Fig. 1C), the comparable magnitudes imply that the fluorine-19-loads in the two single-use cassette-based setups are similar. The Baeyer-Villiger oxidation route employed at OUH was recently implemented on the non-cassette based FlexLab module (iPHASE technologies, Australia) (Huang et al. 2020) leading to batches of [^18^F]**1** with a molar activity in excess of 400 GBq/μmol. The higher molar activity compared to that of the cassette-based approaches reported on here, points to the single-use plastic part as a source of cold fluorine, although, considering the maximum theoretical molar activity of ~ 63 TBq/μmol, fluoride abstraction from the fluoropolymer cyclotron-tubing along with fluoride traces in the oxygen-18 enriched target water provide for the major contributions. Indeed, according to typical supplier specifications at HUH, the natural fluoride content of the target water may be as high as ~ 10 nmol/ml. Generally, the molar activity of [^18^F]**1** for clinical use should be as high as possible, considering a report on serious adverse effects to the subject following an injection of low-molar-activity [^18^F]**1** (*A*_m_ = 6 MBq/μmol) (Koopmans et al. 2005) prepared according to the destannylation procedure described by de Vries et al. (de Vries et al. 1999). Improved GMP compliant procedure was described by Luurtsema et al. in 2017 (Luurtsema et al. 2017).

**Fig. 3:**
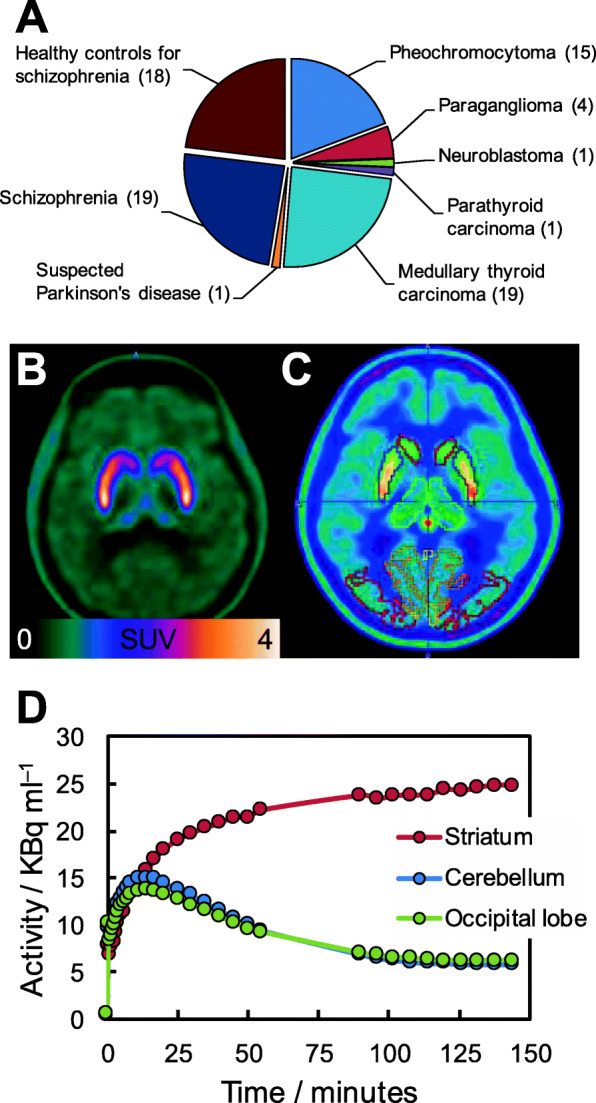
Clinical data from HUH. **A** The composition of the subjects undergoing PET/CT scans with [^18^F]1 from 2017 until March 2020. **B**, **C**, and **D** A dynamic 2.5-h [^18^F]1 scan of the brain in a 22 yo female with schizophrenia, showing high tracer uptake in the striatum with a high target-to-background ratio. For the time activity curves in (**D**), the time scale starts at the start of the first frame

From 2017 until March 2020 a total of 59 patients and 19 healthy volunteers underwent PET/CT scans with [^18^F]**1** at HUH. Some subjects had repeated examinations up to 10 times, totaling 134 performed scans. The composition of the subjects is given in Fig. 3A. Slightly more than half were oncological subjects, of which subjects with either medullary thyroid carcinoma (or suspected recurrent disease) or pheochromocytoma made up the largest subsets. As for the non-oncologic subjects, all but one were participants in a clinical study examining presynaptic dopamine synthesis capacity in the striatum. Figures 3B-D show a dynamic 2.5-h [^18^F]**1** scan of the brain in a patient with schizophrenia (22 yo female). High tracer uptake is seen in the striatum with a high target-to-background ratio. Time activity curves (see Fig. 3D) can be used to model and quantify dopamine synthesis capacity. Further details on the results of the clinical trial will be the subject of future communications.

**Fig. 4:**
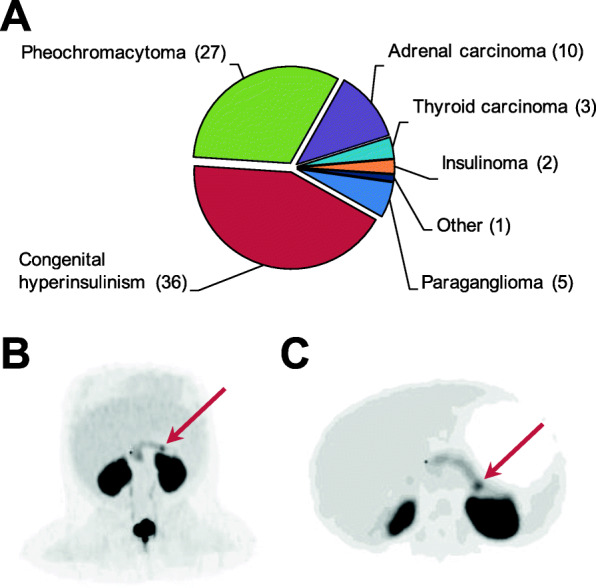
Clinical data from OUH. **A** The composition of the subjects undergoing PET/CT scans with [^18^F]1 from 2017 until 2020. **B** and **C** PET/CT scan of a 3.5-month-old girl suffering from congenital hyperinsulinism. (**B**) gives the maximum intensity projection while (**C**) gives the transverse plane image. The red arrows indicate the minuscule lesion with uptake of [^18^F]1 in the cauda of pancreas

During the same time period at OUH, a total of 84 PET/CT scans with [^18^F]**1** was performed. The composition of the subjects is given in Fig. 4A. Most scans were performed on infants with congenital hyperinsulinism. The PET/CT scan is used to determine if the child has a focal pancreatic lesion as a course of the disorder and hence is candidate for curative surgery. Figures 4B and C, summarize the clinical PET/CT imaging of a girl, 3.5 month of age, admitted to OUH from Sweden with severe hyperinsulinism. The PET/CT scan with [^18^F]**1** showed a minuscule lesion with uptake in the cauda of pancreas. This was surgically enucleated, which lead to immediate rise in blood glucose (on the operating table). In a few days the subject was completely out of medication and declared cured of congenital hyperinsulinism without sign of brain damage. The remainder of PET/CT scans with [^18^F]**1** at OUH were performed on patients with adrenal diseases like pheochromocytomas and carcinomas, and to a lesser degree, paragangliomas and medullary thyroid carcinomas.

At AUH, scans with [^18^F]**1** have been carried out since 1998. Currently, the tracer is used for imaging of dopamine synthesis in patients with Parkinson’s disease as well as tumor diagnostics in patients with pheochromocytoma, paraganglioma, insulinoma, medullar thyroidal cancer, neuroblastoma, and hyperinsulinism. Additionally, PET/CT-scans with [^18^F]**1** are employed in various research projects mainly in the field of neurology. Roughly, 85% of the patients at AUH are scanned on oncology indications, whereas the remainder are scanned on neurological indications. At the introduction of [^18^F]**1** in 1998, however, the ratio between oncological and neurological subjects was in reverse, with Parkinsonians patients making up the main group of subjects. This interestingly reflects a shift in the application of [^18^F]**1** as PET tracer over the time period considered and again attests to the versatility of [^18^F]**1** for a wide range of indications.

## Conclusion

[^18^F]**1** can be reliably produced in multidose quantities using either of the methods discussed here. However, the electrophilic substitution method employed at AUH is disfavored compared to the two nucleophilic substitution approaches primarily due to the demanding operation and maintenance of the fluorine-gas cyclotron targets. However, the low molar activity resulting from the addition of carrier fluorine is also a concern for clinical applications, which further favors the no-carrier added nucleophilic synthesis methods. The enantioselective alkylation approach to [^18^F]**1** employed at HUH is clearly favored in terms of radiochemical yield. The comprehensive setup including harsh chemicals, however, introduces maintenance considerations which are less pertinent to the purely SPE cartridge-based experimental setup for the Bayer-Villiger oxidation approach employed at OUH. The choice between the two nucleophilic methods consequently comes down to a trade-off between high yield and ease of use.

## Supplementary Information

**Additional file 1: Supplemental Table 1**. AUH PET center has three cyclotrons for PET isotope production as its disposal, and thus, three different paths for the production of [^18^F]F_2_ are available. The cyclotron production parameters for the three different cyclotrons are summarized below. All three procedures involve proton beam irradiation of [^18^O]O_2_ gas, followed by cryogenic recovery of the [^18^O]O_2_ gas, and finally a consecutive proton irradiation on a noble gas containing ^19^F_2_ for F-18 recovery. All targets are evacuated for 30 min prior to usage in order to ensure proper operation. For the IBA setup, consecutive filling and emptying with Ne gas is necessary prior to evacuation. **Supplemental Fig. 1**. Setup for production of [^18^F]1 on the custom-build synthesis platform at AUH. The platform consists of a series of 12 V three-way valves, an electrical heater, a vacuum membrane pump, a pneumatically actuated HPLC injector with 5 ml stainless steel loop, HPLC pump, and a photodiode radio detector. The flow of [^18^F]F_2_ is adjusted by a needle valve. Where needed, overpressure is generated by helium. The fluorination reactor and the hydrolysis vessel are conventional Pyrex glassware. For the other containers, conventional single use sterile medical plastic materials are used. **Supplemental Fig. 2**. GUI of the LabVIEW application used for controlling the synthesis platform at AUH. **Supplemental Fig. 3**. Flowchart for the production of [^18^F]1 at AUH. **Supplemental Fig. 4**. Tracerlab MX layout within the synthesis unit control software at OUH. **Supplemental Fig. 5.** Flowchart for the synthesis of [^18^F]1 at OUH. **Supplemental Fig. 6**. Setup of the Tracerlab MX at OUH just before start of synthesis with vial contents and SPE cartridge types given. **Supplemental Fig. 7**. Trasis AllinOne layout within the synthesis unit control software at HUH. **Supplemental Fig. 8**. Setup of the Trasis AiO at HUH just before the start of synthesis with all the reagent vials/containers labelled. Note that the alkylation vial (vial D) is hidden behind the WFI IV bag. The contents of the different vials/syringes are as follows: Vial A contains QMA eluent (K_222_ and K_2_CO_3_ in methanol), vial B contains precursor 7 in DMF, syringe C contains 45% potassium hydroxide, vial D contains the Schiff base *N*-(diphenylmethylene)glycine *tert*-butyl ester and the phase-transfer catalyst as solids, vial E contains dichloromethane, vial F contains solid NaBH_4_, vial G contains ethanol, and vial H contains hydroiodic acid. An IV bag containing water for injections (WFI) is installed in position I, vial J contains solid ascorbic acid, and vial K contains a mixture of citric acid and trisodium citrate dihydrate in WFI (i.e. sterile citrate buffer). **Supplemental Table 2**. The specifications for preparations of [^18^F]1 at AUH. **Supplemental Table 3**. The specifications for preparations of [^18^F]1 at OUH. **Supplemental Table 4**. The specifications for preparations of [^18^F]1 at HUH. **Supplemental Fig. 9**. UV- and radiodetected HPLC chromatograms of a batch of [^18^F]1 at AUH. **Supplemental Fig. 10**. UV- and radiodetected HPLC chromatograms for the determination of the enantiomeric purity of a batch of [^18^F]1 at AUH. **Supplemental Fig. 11**. UV- (283 nm) and radioanalytical HPLC trace for a batch of [^18^F]1 produced at OUH. The UV-trace is given in blue, while the radiodetected chromatogram is given in red. **Supplemental Fig. 12**. UV- (280 nm) and radioanalytical HPLC trace for a batch of [^18^F]1 produced at HUH. The UV-trace is given in green, while the radiodetected chromatogram is given in purple. **Supplemental Fig. 13**. Radiodetected HPLC chromatogram for the determination of the enantiomeric purity of a batch of [^18^F]1 at HUH. The batch is the same as the one used in Supplemental Fig. 12. **Supplemental Fig. 14**. The alkylation vial (vial D) before (left) and after (right) addition of DCM. Note the powdered solids sticking to the sides and bottom of the vial before addition of solvent. After addition of DCM, several specks of suspended solids can be seen sticking to the bottom of the vial.

## Data Availability

The datasets used and/or analysed during the current study are included in this published article and its supplementary information files.

## Abbreviations

6-[^18^F]F-l-DOPA: 3,4-dihydroxy-6-^18^F-l-phenylalanine
AUH: Aarhus University Hospital
AIO: AllinOne
CT: Computed tomography
DCM: Dichloromethane
EDTA: Ethylenediaminetetraacetic
GMP: Good manufacturing practice
HPLC: High-performance liquid chromatography
HUH: Herlev University Hospital
*m*-CBPA: *meta*-chloroperoxybenzoic
n.d.c.: Non-decay corrected
OOS: Out-of-specifications
OUH: Odense University Hospital
PET: Positron Emission Tomography
QC: Quality control
RCY: Radiochemical yield
TBA: Tetra(*n*-butyl)ammonium
YO: Year old

## References

[CR1] Adam MJ, Jivan S (1988). Synthesis and purification of l-6-[^18^F]Fluorodopa. Appl Radiat Isot.

[CR2] Blomberg BA, Moghbel MC, Saboury B, Stanley CA, Alavi A (2013). The value of radiologic interventions and ^18^F-DOPA PET in diagnosing and localizing focal congenital Hyperinsulinism: systematic review and meta-analysis. Mol Imaging Biol.

[CR3] Chirakal R, Firnau G, Schrobilgen GJ, McKay J, Garnett ES (1984). The synthesis of [^18^F]xenon difluoride from [^18^F] fluorine gas. Int J Appl Radiat Isot.

[CR4] de Vries EFJ, Luurtsema G, Brüssermann M, Elsinga PH, Vaalburg W (1999). Fully automated synthesis module for the high yield one-pot preparation of 6-[^18^F]fluoro-l-DOPA. Appl Radiat Isot.

[CR5] Deroose CM, Hindié E, Kebebew E, Goichot B, Pacak K, Taïeb D (2016). Molecular imaging of Gastroenteropancreatic neuroendocrine tumors: current status and future directions. J Nucl Med.

[CR6] Dolle F, Demphel S, Hinnen F, Fournier D, Vaufrey F, Crouzel C (1998). 6-[^18^F]Fluoro-*L*-DOPA by Radiofluorodestannylation: a short and simple synthesis of a new labelling precursor. J Label Compd Radiopharm.

[CR7] Elkashef AM, Doudet D, Bryant T, Cohen RM, Li S-H, Wyatt RJ (2000). 6-^18^F-DOPA PET study in patients with schizophrenia. Psychiatry Res Neuroimaging.

[CR8] Ernst M, Zametkin AJ, Jons PH, Matochik JA, Pascualvaca D, Cohen RM (1999). High presynaptic dopaminergic activity in children with Tourette's disorder. J Am Acad Child Adolesc Psychiatry.

[CR9] Firnau G, Chirakal R, Garnett ES (1984). Aromatic Radiofluorination with [^18^F]fluorine gas: 6-[^18^F]Fluoro-l-Dopa. J Nucl Med.

[CR10] Firnau G, Chirakal R, Sood S, Garnett S (1980). Aromatic fluorination with xenon difluoride: l-3,4-dihydroxy-6-fluoro-phenylalanine. Can J Chem.

[CR11] Garnett ES, Firnau G, Nahmias C (1983). Dopamine visualized in the basal ganglia of living man. Nature..

[CR12] Garnett S, Firnau G, Nahmias C, Chirakal R (1983). Striatal dopamine metabolism in living monkeys examined by positron emission tomography. Brain Res.

[CR13] Huang Y-Y, Poniger S, Tsai C-L, Tochon-Danguy HJ, Ackermann U, Yen R-F (2020). Three-step two-pot automated production of NCA [^18^F]FDOPA with FlexLab module. Appl Radiat Isot.

[CR14] Jääskeläinen SK, Rinne JO, Forssell H, Tenovuo O, Kaasinen V, Sonninen P (2001). Role of the dopaminergic system in chronic pain – a fluorodopa-PET study. Pain..

[CR15] Koopmans KP, Brouwers AH, De Hooge MN (2005). Carcinoid crisis after injection of 6-^18^F-Fluorodihydroxyphenylalanine in a patient with metastatic carcinoid. J Nucl Med.

[CR16] Kushchayev SV, Kushchayeva YS, Tella SH, Glushko T, Pacak K, Teytelboym OM (2019). Medullary thyroid carcinoma: an update on imaging. J Thyroid Res.

[CR17] Libert LC, Franci X, Plenevaux AR, Ooi T, Maruoka K, Luxen AJ (2013). Production at the curie level of no-carrier-added 6-^18^F-Fluoro-l-Dopa. J Nucl Med.

[CR18] Luurtsema G, Boersma HH, Schepers M, de Vries AMT, Maas B, Zijlma R (2017). Improved GMP-compliant multi-dose production and quality control of 6-[^18^F]fluoro- l-DOPA. EJNMMI Radiopharm Chem.

[CR19] Minn H, Kauhanen S, Seppänen M, Nuutila P (2009). ^18^F-FDOPA: a multiple-target molecule. J Nucl Med.

[CR20] Mossine AV, Tanzey SS, Brooks AF, Makaravage KJ, Ichiishi N, Miller JM (2020). Synthesis of high-molar-activity [^18^F]6-fluoro- l-DOPA suitable for human use via cu-mediated fluorination of a BPin precursor. Nat Protoc.

[CR21] Nickles RJ, Daube ME, Ruth TJ (1984). An ^18^O_2_ target for the production of [^18^F]F_2_. Int J Appl Radiat Isot.

[CR22] Nickles RJ, Hichwa RD, Daube ME, Hutchins GD, Congdon DD (1983). An ^18^O_2_-target for the high yield production of ^18^F-fluoride. Int J Appl Radiat Isot.

[CR23] Nieminen-von Wendt TS, Metsähonkala L, Kulomäki TA (2004). Increased presynaptic dopamine function in Asperger syndrome. NeuroReport..

[CR24] O'Donnell MJ, Boniece JM, Earp SE (1978). The synthesis of amino acids by phase-transfer reactions. Tetrahedron Lett.

[CR25] Orlovskaya V, Fedorova O, Kuznetsova O, Krasikova R (2020). Cu-mediated radiofluorination of aryl pinacolboronate esters: alcohols as solvents with application to 6- l-[^18^F]FDOPA synthesis. Eur J Org Chem.

[CR26] Pretze M, Franck D, Kunkel F, Foßhag E, Wängler C, Wängler B (2017). Evaluation of two nucleophilic syntheses routes for the automated synthesis of 6-[^18^F]fluoro-l-DOPA. Nucl Med Biol.

[CR27] Pretze M, Wängler C, Wängler B (2014). 6-[^18^F]Fluoro-l-DOPA: a well-established Neurotracer with expanding application Spectrum and strongly improved Radiosyntheses. Biomed Res Int.

[CR28] Sioka C, Fotopoulos A, Kyritsis AP (2010). Recent advances in PET imaging for evaluation of Parkinson’s disease. Eur J Nucl Med Mol Imaging.

[CR29] Tredwell M, Preshlock SM, Taylor NJ, Gruber S, Huiban M, Passchier J (2014). A general copper-mediated nucleophilic ^18^F fluorination of arenes. Angew Chem Int Ed.

[CR30] Wagner FM, Ermert J, Coenen HH (2009). Three-step "one-pot" radiosynthesis of 6-fluoro-3,4-dihydroxy-l-phenylalanine by isotopic exchange. J Nucl Med.

[CR31] Xiao J, Jin Y, Nie J, Chen F, Ma X (2019). Diagnostic and grading accuracy of ^18^F-FDOPA PET and PET/CT in patients with gliomas: a systematic review and meta-analysis. BMC Cancer.

