# Supplementary material for: GMP production of 6-[18F]Fluoro-l-DOPA for PET/CT imaging by different synthetic routes: a three center experience
Source: EJNMMI Radiopharm Chem. 2021 Jun 12;6:21. doi: 10.1186/s41181-021-00135-y (PMC8197687; doi:10.1186/s41181-021-00135-y)
Supplement: Supplementary file 1 — Additional file 1: Supplemental Table 1. AUH PET center has three cyclotrons for PET isotope production as its disposal, and thus, three different paths for the production of [18F]F2 are available. The cyclotron production parameters for the three different cyclotrons are summarized below. All three procedures involve proton beam irradiation of [18O]O2 gas, followed by cryogenic recovery of the [18O]O2 gas, and finally a consecutive proton irradiation on a noble gas containing 19F2 for F-18 recovery. All targets are evacuated for 30 min prior to usage in order to ensure proper operation. For the IBA setup, consecutive filling and emptying with Ne gas is necessary prior to evacuation. Supplemental Fig. 1. Setup for production of [18F]1 on the custom-build synthesis platform at AUH. The platform consists of a series of 12 V three-way valves, an electrical heater, a vacuum membrane pump, a pneumatically actuated HPLC injector with 5 ml stainless steel loop, HPLC pump, and a photodiode radio detector. The flow of [18F]F2 is adjusted by a needle valve. Where needed, overpressure is generated by helium. The fluorination reactor and the hydrolysis vessel are conventional Pyrex glassware. For the other containers, conventional single use sterile medical plastic materials are used. Supplemental Fig. 2. GUI of the LabVIEW application used for controlling the synthesis platform at AUH. Supplemental Fig. 3. Flowchart for the production of [18F]1 at AUH. Supplemental Fig. 4. Tracerlab MX layout within the synthesis unit control software at OUH. Supplemental Fig. 5. Flowchart for the synthesis of [18F]1 at OUH. Supplemental Fig. 6. Setup of the Tracerlab MX at OUH just before start of synthesis with vial contents and SPE cartridge types given. Supplemental Fig. 7. Trasis AllinOne layout within the synthesis unit control software at HUH. Supplemental Fig. 8. Setup of the Trasis AiO at HUH just before the start of synthesis with all the reagent vials/containers labelled. Note that [file 41181_2021_135_MOESM1_ESM.docx]

Supplemental Table 1 AUH PET center has three cyclotrons for PET isotope production as its disposal, and thus, three different paths for the production of [^18^F]F_2_ are available. The cyclotron production parameters for the three different cyclotrons are summarized below. All three procedures involve proton beam irradiation of [^18^O]O_2_ gas, followed by cryogenic recovery of the [^18^O]O_2_ gas, and finally a consecutive proton irradiation on a noble gas containing ^19^F_2_ for F-18 recovery. All targets are evacuated for 30 minutes prior to usage in order to ensure proper operation. For the IBA setup, consecutive filling and emptying with Ne gas is necessary prior to evacuation.

|  | GE PETtrace 600 | GE PETtrace 800 | IBA Cyclone 18/18 |
| --- | --- | --- | --- |
| Target gas O-18 gas | [^18^O]O_2_ > 98% | [^18^O]O_2_ > 98% | [^18^O]O_2_ > 98% |
| Target body | Aluminium | Aluminium | Aluminium |
| Filling pressure | 10 bar | 10 bar | 20 bar |
| Beam current | 40 µA | 40 µA | Up to 40 µA |
| Standard irradiation time | 60 min | 60 min | 60 min |
| Target gas for F-18 recovery | Ar containing He-F_2_^‡^ | Ar containing He-F_2_^‡^ | Ne containing 1% F_2_ |
| Filling pressure | 10 bar | 10 bar | 20 bar |
| Beam current | 30 µA | 30 µA | Up to 40 µA^†^ |
| Standard irradiation time | 20 min | 20 min | 20 min |
| Specified [^18^F]F_2_ (EOB) | 45 GBq | 60 GBq | 45 GBq |

^†^Beam current may vary according to maximum allowed target pressure. ^‡^Final F_2_-content 0.5%.

**Radiosynthesis at AUH:** Following the second bombardment of the “double-shoot” method (see Supplemental Table 1 for details), [^18^F_2_]F_2_ is delivered to the synthesis platform within approximately 5 minutes at a flow rate of 300 ml/min through a solution of 6-trimethylstannyl-l-DOPA (50 mg, 0.14 mmol) in anhydrous CHCl_3_ (5 ml) at RT. Upon completion of the delivery, the reaction mixture is passed through two Silica Sep-Pak Classic solid-phase extraction (SPE) cartridges, loaded with sodium thiosulfate (20 mg), into the hydrolysis vessel using negative pressure. The SPE cartridges are washed with anhydrous Et_2_O (15 ml), and the combined chloroform and ether solutions are evaporated to dryness *in vacuo* at elevated temperatures. The residue is treated with hydriodic acid (1.8 ml, 57%, stabilized) and heated at 145 °C for 20 min. The solution is subsequently allowed to cool for 2 min and then added sterile aqueous NaOH (3 ml, 3 m). The partially neutralized solution is purified by semi-preparative radio-HPLC as described in the main text.


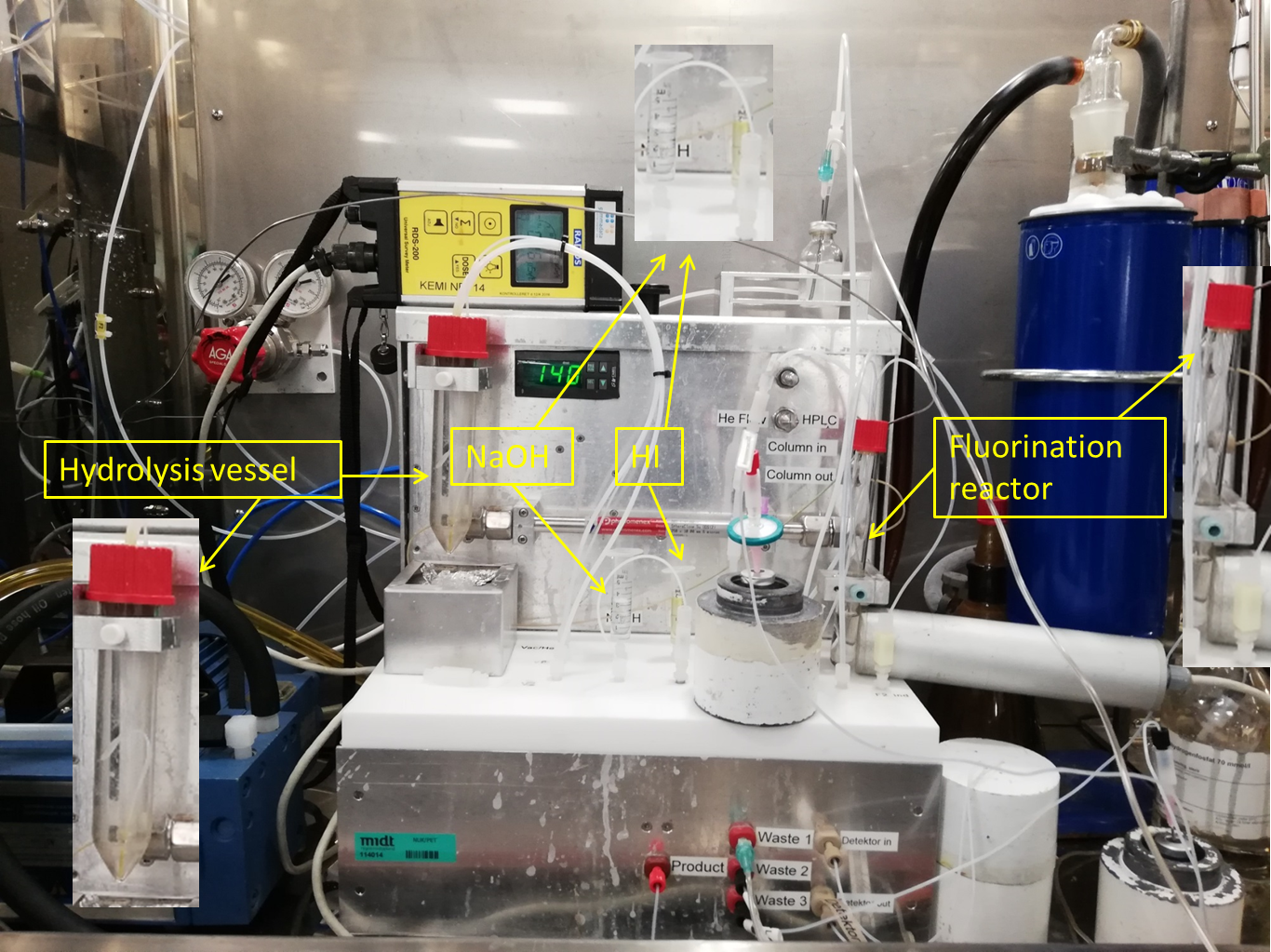


Supplemental Figure 1 Setup for production of [^18^F]1 on the custom-build synthesis platform at AUH. The platform consists of a series of 12 V three-way valves, an electrical heater, a vacuum membrane pump, a pneumatically actuated HPLC injector with 5 ml stainless steel loop, HPLC pump, and a photodiode radio detector. The flow of [^18^F]F_2_ is adjusted by a needle valve. Where needed, overpressure is generated by helium. The fluorination reactor and the hydrolysis vessel are conventional Pyrex glassware. For the other containers, conventional single use sterile medical plastic materials are used.


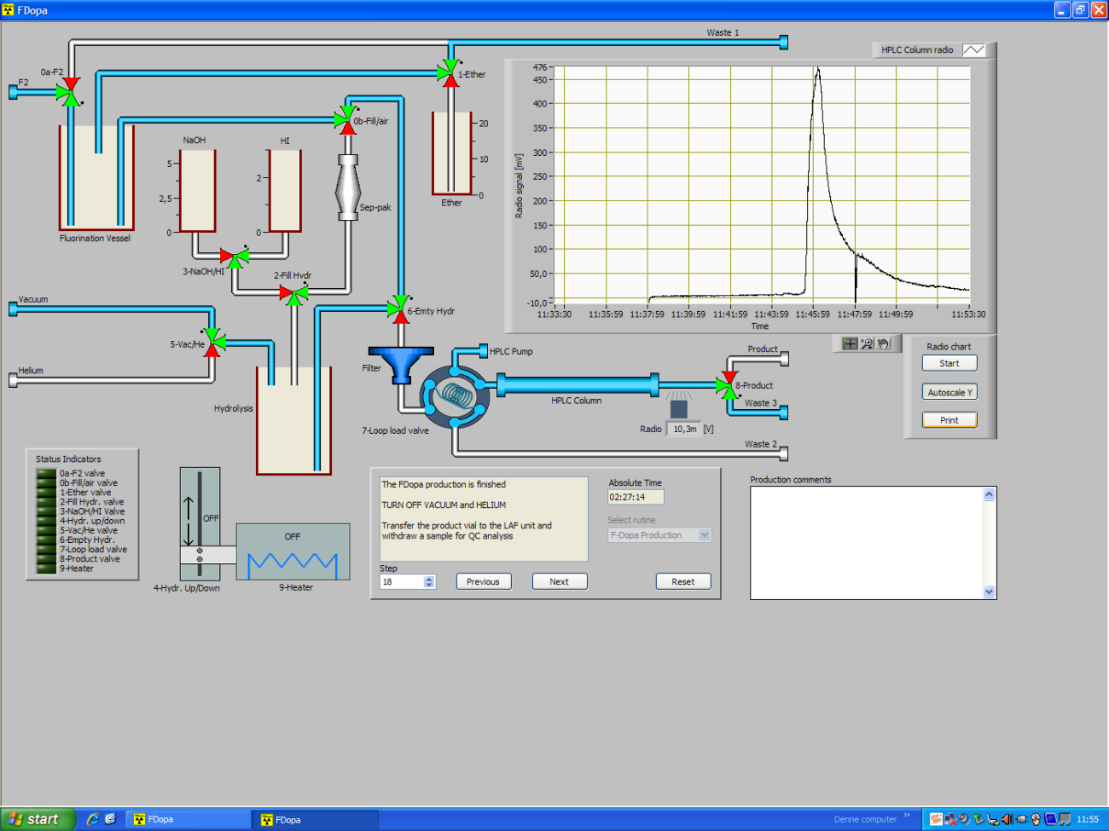


Supplemental Figure 2 GUI of the LabVIEW application used for controlling the synthesis platform at AUH.


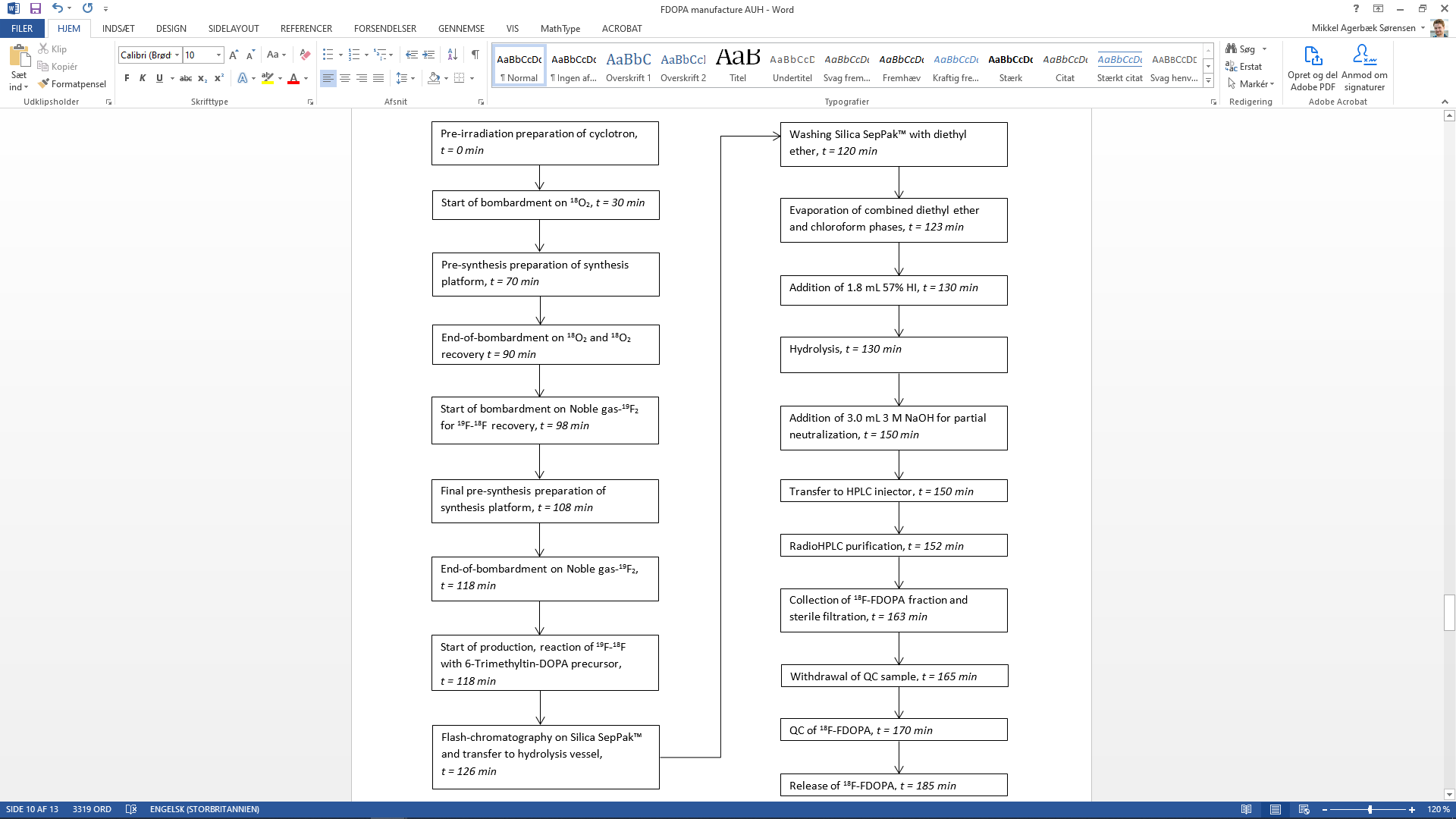


Supplemental Figure 3 Flowchart for the production of [^18^F]1 at AUH.


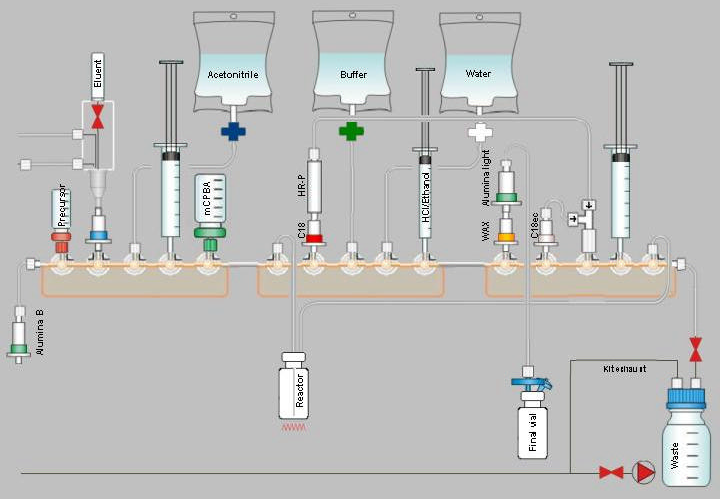


Supplemental Figure 4 Tracerlab MX layout within the synthesis unit control software at OUH.


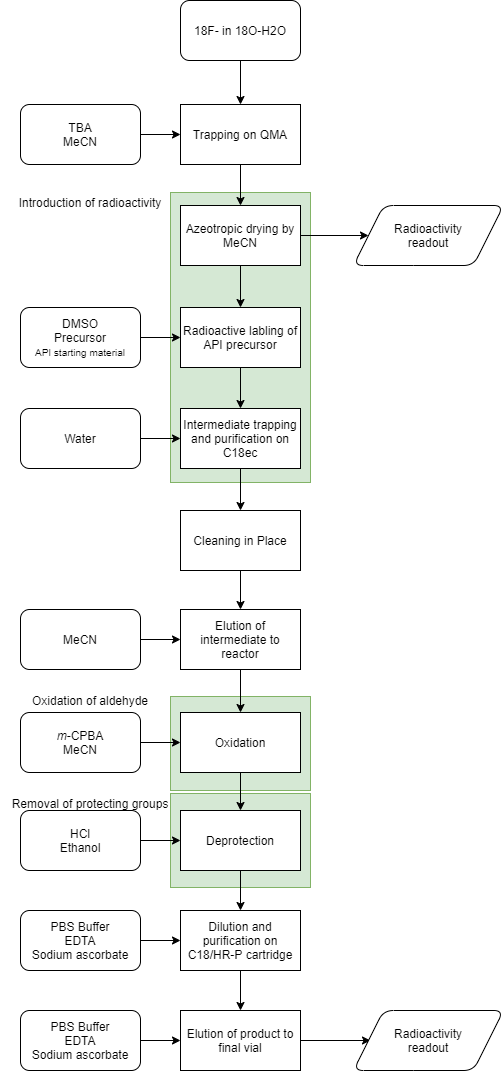


Supplemental Figure 5 Flowchart for the synthesis of [^18^F]1 at OUH.


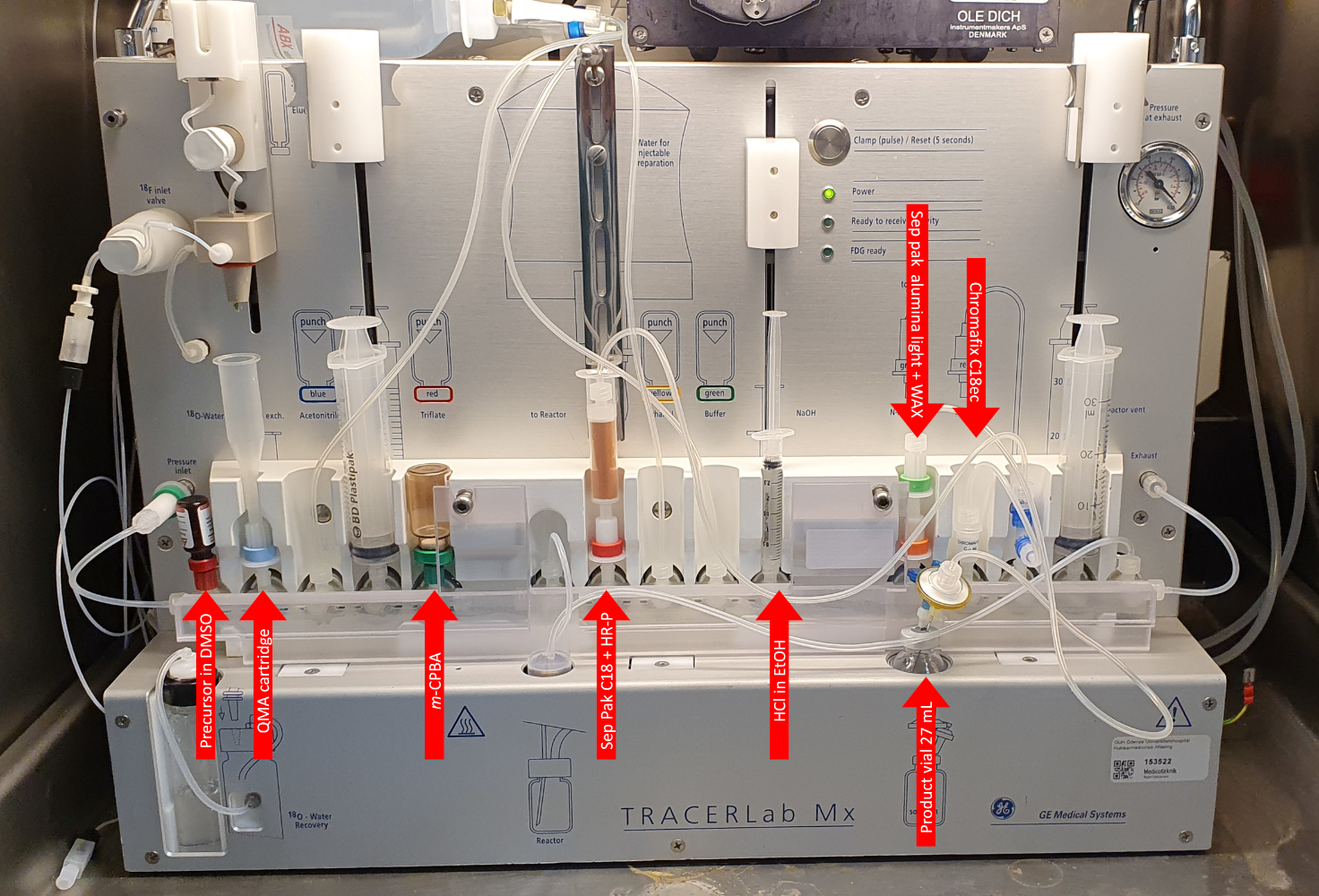


Supplemental Figure 6 Setup of the Tracerlab MX at OUH just before start of synthesis with vial contents and SPE cartridge types given.

*
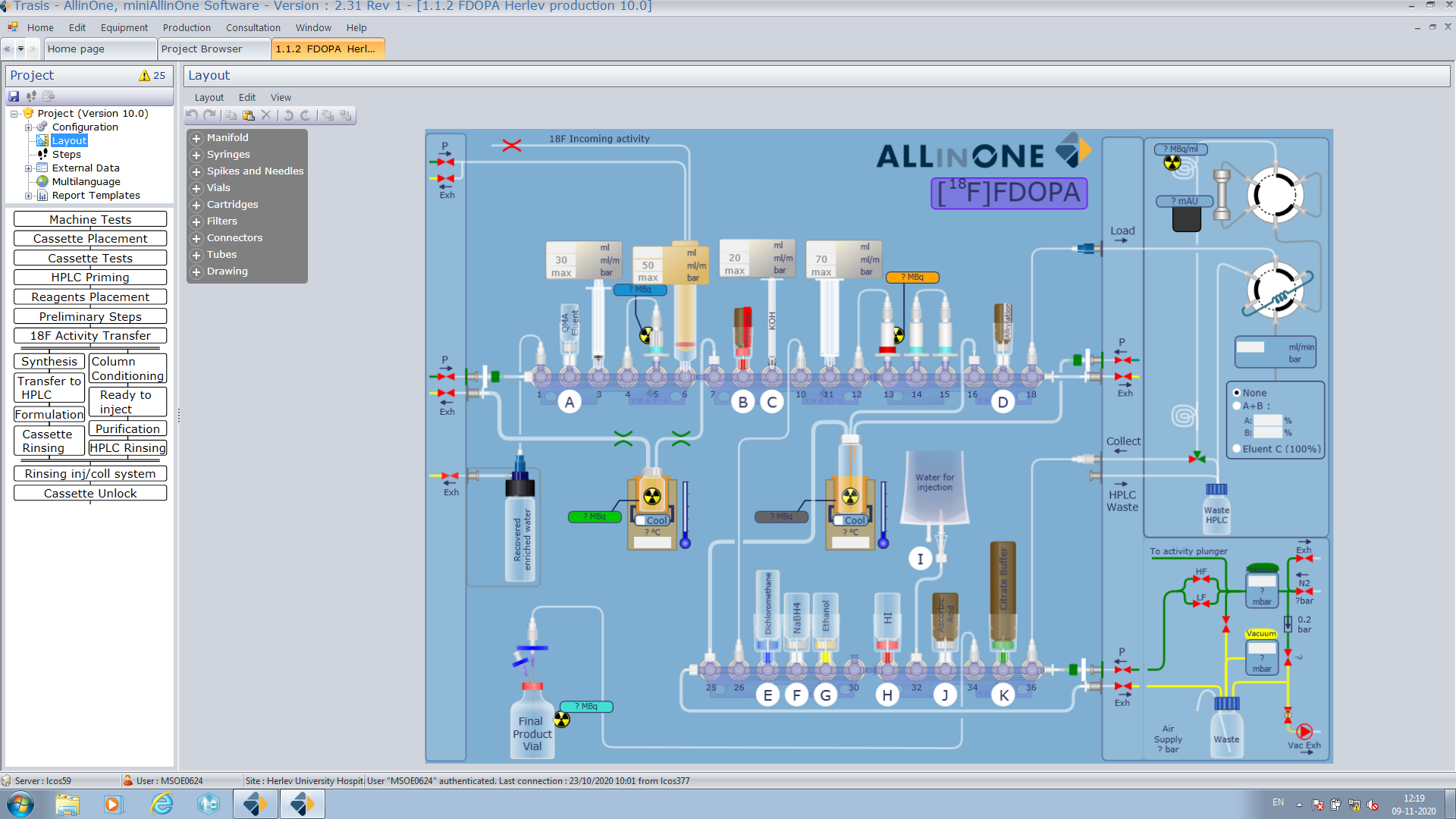
*

Supplemental Figure 7 Trasis AllinOne layout within the synthesis unit control software at HUH.

Supplemental Figure 8 Setup of the Trasis AiO at HUH just before the start of synthesis with all the reagent vials/containers labelled. Note that the alkylation vial (vial D) is hidden behind the WFI IV bag. The contents of the different vials/syringes are as follows: Vial A contains QMA eluent (K_222_ and K_2_CO_3_ in methanol), vial B contains precursor 7 in DMF, syringe C contains 45% potassium hydroxide, vial D contains the Schiff base *N*-(diphenylmethylene)glycine *tert*-butyl ester and the phase-transfer catalyst as solids, vial E contains dichloromethane, vial F contains solid NaBH_4_, vial G contains ethanol, and vial H contains hydroiodic acid. An IV bag containing water for injections (WFI) is installed in position I, vial J contains solid ascorbic acid, and vial K contains a mixture of citric acid and trisodium citrate dihydrate in WFI (i.e. sterile citrate buffer).

Supplemental Table 2 The specifications for preparations of [^18^F]1 at AUH.

|  | Specification |
| --- | --- |
| Volume | 5-10 ml |
| Appearance | Colorless, no visible particles |
| pH | 4.0-5.0 |
| [^19^F]**1** | < 2.0 mg/ml |
| l-DOPA | < 1.0 mg/ml |
| 6-OH-DOPA | < 10.0 µg/ml |
| Radiochemical purity | ≥ 95% |
| Enantiomeric purity (l-isomer) | > 95% |
| Sn content | < 0.5 µg/ml |
| *T*_1/2_ | 105-115 min |
| *γ*-spectrometry | 511 + 1022 keV |
| Endotoxins | < 17.5 EU/ml |
| Sterility | Sterile |
| Shelf-life**^†^** | 6 h |

^†^Based on appearance, radiochemical purity, and pH.

Supplemental Table 3 The specifications for preparations of [^18^F]1 at OUH.

|  | Specification |
| --- | --- |
| Radioactivity | 50-12190 MBq |
| Batch volume, V | 24 ml |
| Patient max. volume, *V* | 6 ml |
| Appearance | Clear & colorless |
| pH | 4.0-6.0 |
| Radiochemical purity | ≥ 95% |
| Radionuclidic purity | ≥ 99.9% |
| Sterility | No growth |
| Enantiomeric purity (l-isomer) | ≥ 96% |
| Endotoxins | ≤ 29 EU/ml (< 175/*V*) |
| [^18^F]Fluoride | ≤ 5% |
| Ethanol | ≤ 7% (v/v) |
| Acetonitrile | ≤ 683 ppm (≤ 4.1 mg/*V*) |
| DMSO | ≤ 0.5% (v/v) |
| [^19^F]**1** | ≤ 16.6 µg/ml (≤ 100 µg/*V*) |
| 6-OH-DOPA | ≤ 8.3 µg/ml (≤ 50 µg/*V*) |
| TBA | ≤ 0.43 mg/ml (≤ 2.6 mg/*V*) |
| *T*_1/2_ | 105-115 min |
| *γ*-spectrometry | 511 KeV |
| Shelf-life | EOS + 4h |

Supplemental Table 4 The specifications for preparations of [^18^F]1 at HUH.

|  | Specification |
| --- | --- |
| Appearance | Clear and colorless |
| pH | 4.0-5.5 |
| Ethanol | ≤ 0.5% |
| Acetic acid | ≤ 0.5% |
| Methanol | ≤ 3000 ppm |
| Dimethylformamide | ≤ 880 ppm |
| Dichloromethane | ≤ 600 ppm |
| Radiochemical purity | ≥ 95% |
| [^18^F]Fluoride | ≤ 2.0% |
| Enantiomeric purity (l-isomer) | ≥ 95% |
| [^19^F]**1** | ≤ 40.0 µg/ml |
| Kryptofix 222 | < 88 µg/ml |
| Endotoxins | Complies with Ph. Eur. 2.6.1 |
| *γ*-spectrometry | 511 + 1022 keV |
| Radioactivity concentration EOS | 111-1644 MBq/ml |
| *T*_1/2_ | 105-115 min |
| Shelf-life | EOS + 6h |
| Sterility | Complies with Ph. Eur. 2.6.1 |
| Radionuclidic purity | ≥ 99.9% |
| Final volume | 25 ml |

## QC procedures

**AUH:** The radiochemical and chemical purity is determined by HPLC using a Spherisorb ODS column (250 × 10 mm, Phenomenex) and 70 mm NaH_2_PO_4_ buffer as eluent at a flow rate of 1 ml/min. The eluate is analyzed by UV- (λ = 280 nm) and radiodetection (NaI scintillation detector). The radiochemical purity is determined by the area of the peak corresponding to [^18^F]**1** compared to the total area of the radiochromatogram, whilst the tests for l-DOPA, 6-OH-DOPA, and [^19^F]**1** are performed as limit tests based on the respective areas in the UV-detected chromatogram compared to the peak areas obtained from individual solutions containing the limit amounts (see Supplemental Table 2 for specification) analyzed prior to QC. Representative chromatograms are given in Supplemental Fig. 9.


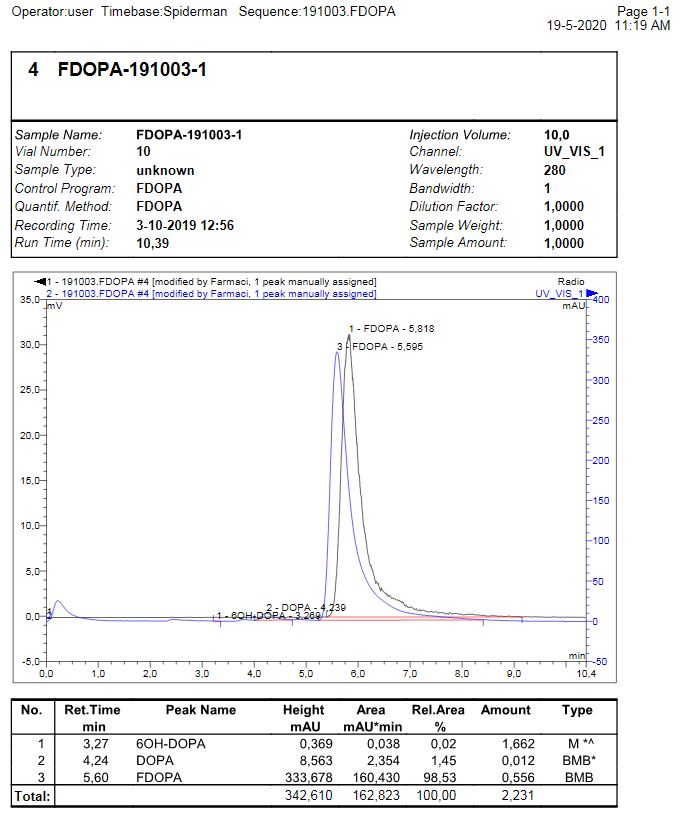


Supplemental Figure 9 UV- and radiodetected HPLC chromatograms of a batch of [^18^F]1 at AUH.

pH is determined by pH-strips, the integrity of the sterilizing filter by a pressure hold test, and the appearance and volume by visual inspection. On revalidation basis (every 10th batch or every 6 months, whichever comes first) additional QC tests for bacterial endotoxins, sterility (additional samples are tested for sterility on a randomly picked basis), stability, half-life, radionuclidic purity (by γ-spectrometry), and enantiomeric purity are performed. The content of endotoxins is determined using an Endosafe PTS endotoxin testing unit (Charles River) on single use cartridges (PTS 20F 0.05-5 EU/ml) applying a dilution factor of 50, using water for injection as diluent. Radionuclidic purity is determined using a DSA-1000 MCA from Canberra equipped with a Cryo-Pulse 5 electrically refrigerated cryostat, a Von Gahlen 98-11B lead castle, a sample holder, and the Genie-2000 software. The multichannel analyzer is calibrated using a ^133^Ba- and a ^152^Eu-source, both in the hundreds of kBq-range. Half-life is determined applying a Capintec CRC-PC Smart Chamber – Dose Calibrator, using software of in-house design. For stability testing after 6 hours besides visual inspection and pH determination, chemical and radiochemical purity are re-tested, applying the regular QC conditions.

The enantiomeric purity is determined by HPLC using a DAICEL Crownpak CR(+) column and aqueous HClO_4_ (pH = 2) as eluent at a flow rate of 0.7 ml/min. The eluate is analyzed by UV- (λ = 280 nm) and radiodetection (NaI scintillation detector). A reference sample containing 6-F-d,l-DOPA is analyzed prior to QC, and the enantiomeric purity is determined by comparison of the peak areas in the radiochromatogram assigned to [^18^F]6-F-d-DOPA and [^18^F]**1**, respectively. Representative chromatograms are given in Supplemental Fig. 10. Upon receiving a new batch of trimethylstannyl-precursor, enantiomeric purity of [^18^F]**1** is tested by radio-HPLC, and the tin content is determined in a contract laboratory by inductively coupled plasma mass spectrometry (ICP-MS). Note that no tests for residual solvents, namely chloroform and diethyl ether, are performed as the absence of these solvents was proven within process validation.


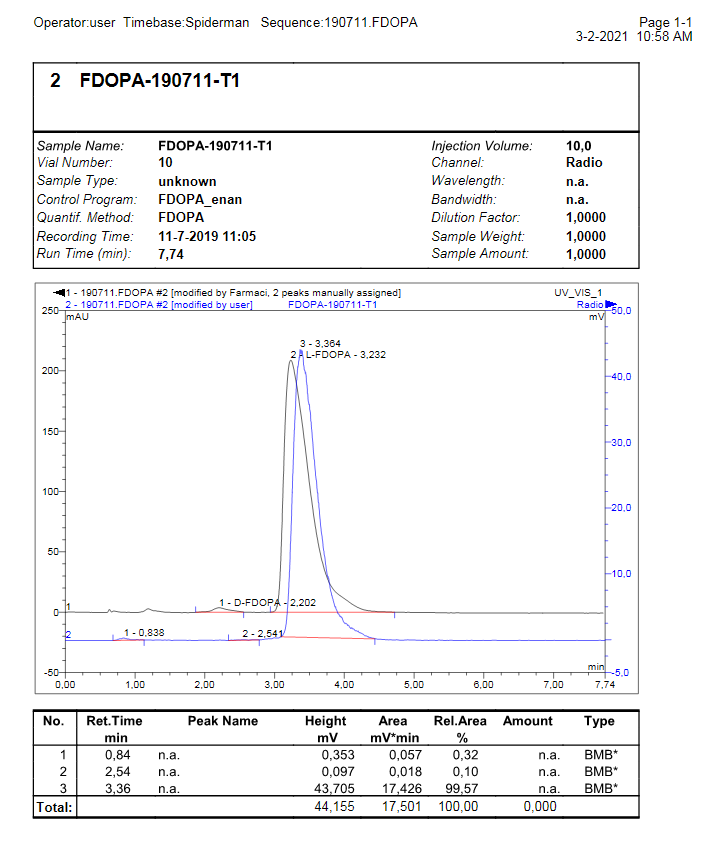


Supplemental Figure 10 UV- and radiodetected HPLC chromatograms for the determination of the enantiomeric purity of a batch of [^18^F]1 at AUH.

**OUH:** The radiochemical and chemical purity is assessed by reversed phase radio-HPLC on an XTerra column (Waters) by the method described in Ph. Eur. monograph 2481 (04/2019). In brief, [^18^F]**1** is analyzed using an acetonitrile gradient in deionized water containing 0.1% trifluoracetic acid at a flow rate of 1 ml/min and UV-detection at 283 nm. A reference sample containing 6-F-d,l-DOPA is analyzed prior to QC. Representative chromatograms are given in Supplemental Figure 11.


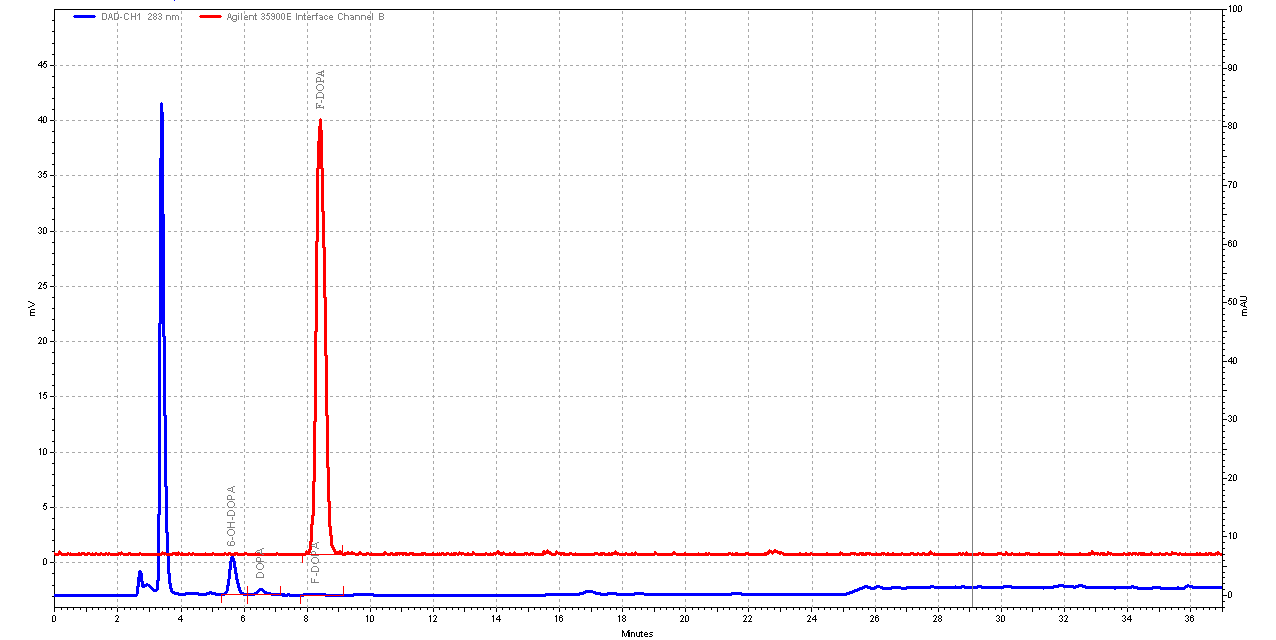


Supplemental Figure 11 UV- (283 nm) and radioanalytical HPLC trace for a batch of [^18^F]1 produced at OUH. The UV-trace is given in blue, while the radiodetected chromatogram is given in red.

Free [^18^F]F^–^ is quantified by reversed phase thin-layer chromatography (RP-TLC) by the method of Füchtner *et al*. and developed in 0.4 M H_2_SO_4_ containing EDTA (5 mm) in which the unbound [^18^F]F^–^ is retained at the origin, while [^18^F]FDOPA elutes with the solvent front (*1*). The RP-TLC is counted on the MiniGITA (Elysia-Raytest) using the Ginastar* software version 6.0. The pH of preparations of [^18^F]**1** is determined on a Mettler Toledo SevenCompact S220 pH-meter with an InLab microelectrode, and residual TBA is determined by a spot test performed simultaneously with a known reference of 33 µg/ml (0.2 mg/*V*) and developed in an iodine chamber following the method described by Kuntszch *et al*. (*2*). The amount of residual ethanol, acetonitrile, and DMSO in each batch of [^18^F]**1** is determined by gas chromatography on a Thermo-Fisher Trace 1310 gas chromatograph equipped with a 1300 Series autoinjector, flame ionization detector (FID), and a Varian capillary column. The program comprises split injection at a starting temperature of 40 °C for 5 min, then 50 °C/min until 240 °C with a constant helium flow of 2.0 ml/min and FID detection at 270 °C. The enantiomeric excess is determined by chiral HPLC every 20^th^ batch and upon reception of new precursor **4**. [^18^F]**1** is analyzed on a DAICEL Crownpak CR(+) (250 × 4.6 mm, 5 µm) column using 10 mm HClO_4_ as eluent at a flow rate of 1 ml/min. The eluate is analyzed by UV-detection at 200 nm. The enantiomeric purity is assessed by comparison with co-injected authentic 6-F-d,l-DOPA. The radionuclidic purity is assessed by γ-spectrometry using a Canberra NaI multichannel analyzer (MCA) running the Genie 2000 software, and by half-life determination on a CRC-15 PET Capintec dose calibrator. Test for sterility and bioburden is performed by contract analysis according to Ph. Eur. 2.6.1. The integrity of the sterilizing filter for each batch is tested before release for clinical application by manual bubble point verification. The content of endotoxins is determined using an Endosafe Nexgen-PTS endotoxin testing unit (Charles River) on single use cartridges (0.05-5 EU/ml) applying a dilution factor of 50 using LAL-water as diluent.

**HUH:** Sterility studies, endotoxin tests, and determination of bioburden (by membrane filtration) are carried out according to the guidelines found in Ph. Eur. 2.6.1. pH-testing is performed using pH-indicator strips from Merck Millipore (pH 4.0 – 7.0), and this colorimetric test has been validated against a potentiometric pH meter. The shelf-life of preparations of [^18^F]**1** is established based on the evaluation of radiochemical and enantiomeric purity (by HPLC) and pH over a period exceeding the shelf-life. The total radioactivity end of synthesis (EOS) is determined using a Veenstra VDC-505 dose calibrator, the performance of which is checked against a ^137^Cs-source in the MBq-range. Half-life measurements are performed on an Elysia-Raytest Isomed 1010 dose calibrator.

Radiochemical and chemical purity of [^18^F]**1** is assessed using a Shimadzu ultra-fast liquid chromatography system equipped with a DGU-20A_5R_ degassing unit, an LC-20AD prominence liquid chromatograph, an SIL-20Aht prominence autosampler, an SPD-M20A prominence diode array detector, an RID-20A refractive index detector, a CBM-20A prominence communications bus module, and a HPLC GM radiodetector (Scansys Laboratorieteknik). QC samples (injection volume 50 µl) are analyzed using an XBridge BEH shield RP18 250 × 4.6 mm column (Waters) with NaH_2_PO_4_-buffer (pH = 2.4) as mobile phase at a flow rate of 1 ml/min. The eluate is detected by a UV- (280 nm) and a radiodetector connected in series. The total run time of the analysis is 15 min. The performance of the HPLC system is verified on a weekly basis by injection of a 100 µg/ml standard solution of 6-F-d,l-DOPA. If a concentration of 100 ± 10 µg/ml (i.e. ± 10%) is recovered, the performance is deemed acceptable. Representative chromatograms are given in Supplemental Fig. 12.

Supplemental Figure 12 UV- (280 nm) and radioanalytical HPLC trace for a batch of [^18^F]1 produced at HUH. The UV-trace is given in green, while the radiodetected chromatogram is given in purple.

Enantiomeric purity of [^18^F]**1** is determined using a Shimadzu ultra-fast liquid chromatography system equipped with a DGU-20A_5_ degassing unit, an LC-20AD prominence liquid chromatograph equipped with a manual injection valve, an SPD-M20A prominence diode array detector, an RID-10A refractive index detector, a CBM-20A prominence communications bus module, and a HPLC radiodetector Model 105S from Carroll & Ramsey Associates. An Astec Chirobiotic T Chiral 250 × 4.6 mm (5 µm) column from Sigma Aldrich is used. The analysis is performed using MeOH/H_2_O/formic acid (700/300/0.2) as mobile phase with a flow rate of 1.0 ml/min for 10 minutes. The injection volume is 20 µl. The eluate is detected by a UV detector (280 nm) and a radiodetector connected in series. A representative chromatogram is given in Supplemental Fig. 13.

Supplemental Figure 13 Radiodetected HPLC chromatogram for the determination of the enantiomeric purity of a batch of [^18^F]1 at HUH. The batch is the same as the one used in Supplemental Fig. 12.

The content of ethanol, DMF, methanol, DCM, and acetic acid in preparations of [^18^F]**1** is determined by gas chromatography using a Shimadzu NEXIS GC-2030 gas chromatograph equipped with a ZB-WAXplus column (30 m × 0.53 mm × 1 µm), an AOC-20i Plus auto injector, an FID at 250 °C, and an AOC-20s Plus autosampler. The injection volume is 0.5 µl (split injection) with a flowrate of 3 ml/min and a run time of 12.5 min. The performance of the GC system is tested on a weekly basis by injecting an aqueous reference solution containing 1326 ppm DCM and a solution containing 1580 ppm DMF, 4836 ppm methanol, 10520 ppm ethanol, and 10500 ppm acetic acid. The performance of the GC system is deemed acceptable if the determined concentrations are within 10% of their theoretical values.

The composition of radioisotopes in preparations of [^18^F]**1** is determined using a DSA-1000 MCA from Canberra equipped with a Cryo-Pulse 5 electrically refrigerated cryostat, a Von Gahlen 98-11B lead castle, a sample holder, and the Genie-2000 software. The multichannel analyzer is calibrated using a ^133^Ba- and a ^152^Eu-source, both in the hundreds of kBq-range.

**
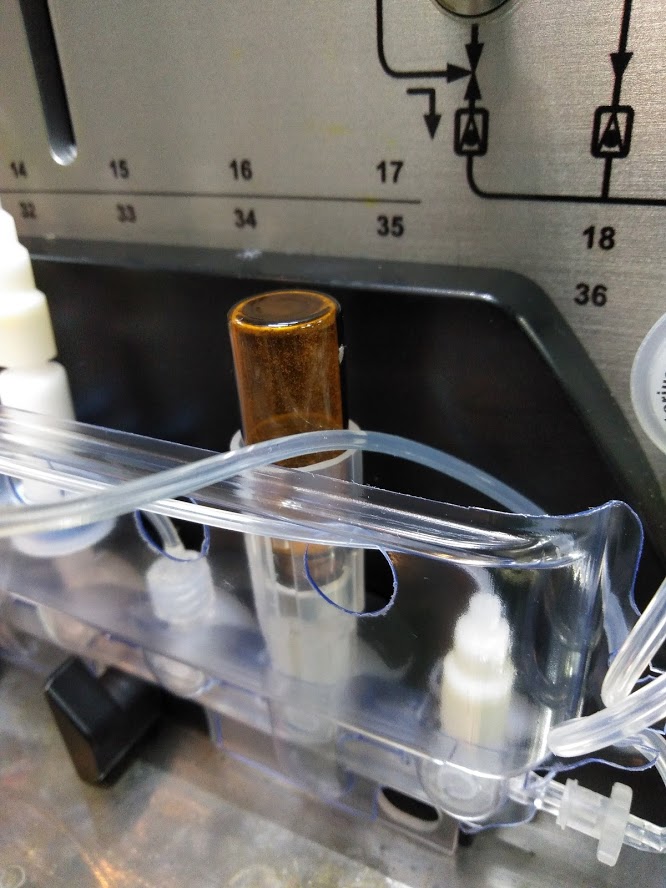
**
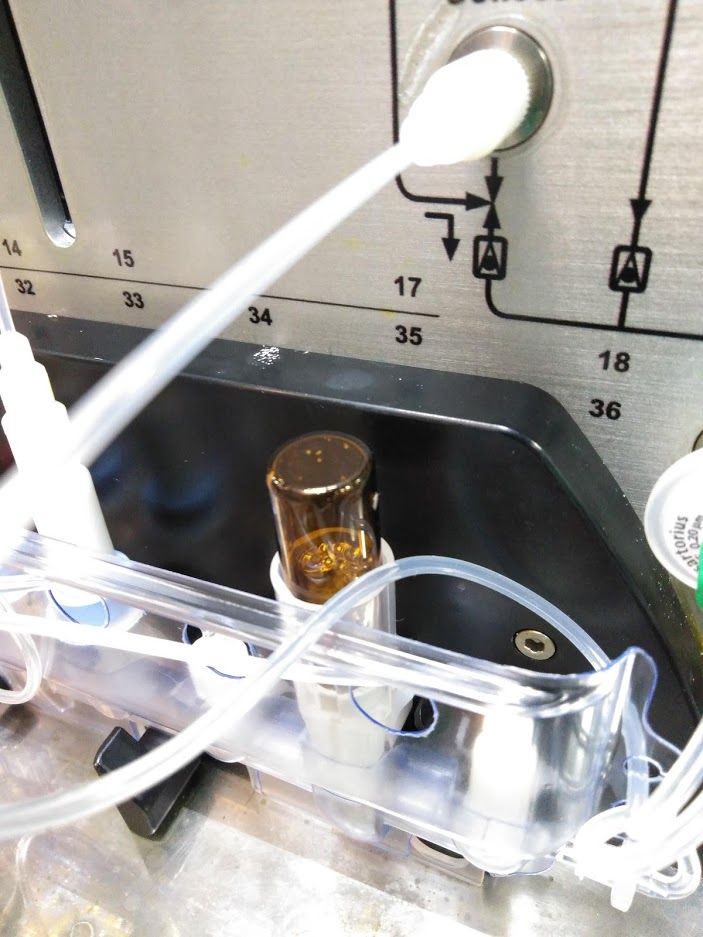


Supplemental Figure 14 The alkylation vial (vial D) before (left) and after (right) addition of DCM. Note the powdered solids sticking to the sides and bottom of the vial before addition of solvent. After addition of DCM, several specks of suspended solids can be seen sticking to the bottom of the vial.

# References

1. Füchtner F, Angelberger P, Kvaternik H, Hammerschmidt F, Simovc BP, Steinbach J. Aspects of 6-[^18^F]fluoro-L-DOPA preparation: precursor synthesis, preparative HPLC purification and determination of radiochemical purity. *Nucl Med Biol.* 2002;29:477-481.

2. Kuntzsch M, Lamparter D, Brüggener N, Müller M, Kienzle GJ, Reischl G. Development and Successful Validation of Simple and Fast TLC Spot Tests for Determination of Kryptofix® 2.2.2 and Tetrabutylammonium in ^18^F-Labeled Radiopharmaceuticals. *Pharmaceuticals.* 2014;7:621-633.
